# Combination or mild single agent chemotherapy for advanced breast cancer? CMF vs epirubicin measuring quality of life.

**DOI:** 10.1038/bjc.1993.74

**Published:** 1993-02

**Authors:** S. C. Fraser, H. J. Dobbs, S. R. Ebbs, L. J. Fallowfield, T. Bates, M. Baum

**Affiliations:** Department of Surgery, Kings College Hospital, London, UK.

## Abstract

Forty patients with advanced breast cancer, randomised to receive CMF or weekly low dose Epirubicin, were evaluated by UICC criteria of response and WHO toxicity criteria, in addition to three QoL instruments: the 'Qualitator' daily diary card, 4 weekly Nottingham Health Profile (NHP) and Linear Analogue Self-Assessment (LASA). Response rates were 58% for CMF and 29% for epirubicin (chi 2 = 3.51, 1 d.f., P > 0.05). Median time to treatment failure was 24 weeks for CMF, 7 weeks for epirubicin (P < 0.05) but survival was similar in both groups. Survival was better for responders than for non-responders (medians 87 and 30 weeks, P = 0.02). CMF caused more objective alopecia (P < 0.001), nausea and vomiting (P < 0.001) and haematological toxicity (P < 0.02). However, QoL measures only recorded a significant difference in energy and pain, influenced primarily by the non-responders in each treatment group but with no difference in overall global scores. Scores for responders, irrespective of treatment, were better to start with (LASA P = 0.001); at 12 weeks, scores had improved (Qualitator P < 0.05; NHP P < 0.05). Scores in non-responders showed no change. In this small study aggressive chemotherapy gave better response and similar survival without impairing Quality of life overall. Detailed QoL measurement should be integral to all cancer chemotherapy trials.


 © Macmillan Press Ltd., 1993

### Combination or mild single agent chemotherapy for advanced breast cancer? CMF *vs* epirubicin measuring Quality of Life

S.C.A. Fraser[1], H.J. Dobbs[2], S.R. Ebbs[3], L.J. Fallowfield[4], T. Bates[5] & M. Baum[6]

[1]*Department of Surgery,* [2]*Department of Radiotherapy and Oncology, Kings College Hospital, Denmark Hill, London SE5 9RS;* [3]*Department of Surgery, Mayday University Hospital, Mayday Road, Thornton Health, Surrey CR7 7YE;* [4]*Cancer Research Campaign Communication and Counselling Research Centre, London Hospital Medical College, Turner St., London E1 2AD;* [5]*Department of Surgery, William Harvey Hospital, Ashford, Kent TN24 0LZ;* [6]*Institute of Cancer Research, Royal Marsden Hospital, Fulham Road, London SW3, UK.*

**Summary** Forty patients with advanced breast cancer, randomised to receive CMF or weekly low dose Epirubicin, were evaluated by UICC criteria of response and WHO toxicity criteria, in addition to three QoL instruments: the 'Qualitator' daily diary card, 4 weekly Nottingham Health Profile (NHP) and Linear Analogue Self-Assessment (LASA). Response rates were 58% for CMF and 29% for epirubicin ($\chi^2 = 3.51$, 1 d.f., $P > 0.05$). Median time to treatment failure was 24 weeks for CMF, 7 weeks for epirubicin ($P < 0.05$) but survival was similar in both groups. Survival was better for responders than for non-responders (medians 87 and 30 weeks, $P = 0.02$). CMF caused more objective alopecia ($P < 0.001$), nausea and vomiting ($P < 0.001$) and haematological toxicity ($P < 0.02$). However, QoL measures only recorded a significant difference in energy and pain, influenced primarily by the non-responders in each treatment group but with no difference in overall global scores. Scores for responders, irrespective of treatment, were better to start with (LASA $P = 0.001$); at 12 weeks, scores had improved (Qualitator $P < 0.05$; NHP $P < 0.05$). Scores in non-responders showed no change. In this small study aggressive chemotherapy gave better response and similar survival without impairing Quality of life overall. Detailed QoL measurement should be integral to all cancer chemotherapy trials.

The treatment of patients with advanced breast cancer using combination chemotherapy can cause significant toxicity without greatly prolonging survival (Powles *et al.*, 1980; A'Hern *et al.*, 1988). Recently, studies have been reported in which low-toxicity regimens (single agent or short term) have achieved palliation without affecting survival (Chlebowski *et al.*, 1989; Harris *et al.*, 1990). For example, Jones has reported a response rate of 43% with epirubicin given with a weekly dose of approximately 20 mg. No significant myelosuppression, and minimal nausea and alopecia resulted (Jones, 1988). Further studies have shown no improvement in response rates by doubling the weekly dose from 20 to 40 mg. There was, however, a considerable increase in toxicity (Ebbs *et al.*, 1989).

There is a danger that such low toxicity regimens may be accepted without adequate comparison with conventional combination cytotoxics. One of the most widely used regimens in advanced breast cancer is the standard Cyclophosphamide, Methotrexate and 5-Fluorouracil (CMF) treatment which achieves response rates of up to 60% (Bonadonna & Van Oosterom, 1983). This was therefore chosen as the control arm of a direct comparison with low-dose weekly epirubicin. As reduced toxicity was central to the development of the low-dose regimen, the trial was planned around detailed measurement of Quality of Life.

### Patients and methods

### *Patients*

Between October 1988 and December 1989, 40 patients with advanced breast cancer attending the Breast clinics at King's College Hospital and the William Harvey Hospital were randomised to receive CMF or epirubicin as first line chemotherapy. Criteria for inclusion were: histologically proven locally advanced disease, rapidly progressing primary disease, metastatic disease failing to respond to hormonal measures, a first recurrence which was visceral, or recurrent disease less than 2 years from primary treatment. Excluded, were postmenopausal women with locally advanced disease suitable for a trial of tamoxifen, those with a significant medical condition or known previous or current cardiovascular disease and patients who had received non-adjuvant chemotherapy. The two groups were evenly matched according to the sites of disease, and menopausal status, although there was a difference in their median ages which was not statistically significant (see Table I).

### *Ethical considerations*

The trial was approved by the ethical committees in both participating hospitals. Written informed consent was obtained from the patients prior to randomisation.

### *Treatment*

All therapy was given in the outpatient clinic by one person. The dose schedules were: (1) Epirubicin 20 mg intravenously, given into fast-running 0.9% saline every 7 days; (2) Cyclo-

**Table I** Characteristics of patients entering study

| | *Epirubicin* | *CMF* |
|---|---|---|
| Number | 21 | 19 |
| Median age | 52 (26-80) | 63 (39-84) |
| Premenopausal | 9 | 4 |
| Postmenopausal | 12 | 15 |
| Sites: soft tissues | 10 | 10 |
| Nodal | 9 | 12 |
| Lung | 6 | 6 |
| Liver | 6 | 6 |
| Bone | 9 | 9 |
| QoL medians and ranges | | |
| NHP | 129 (13-308) | 91 (13-350) |
| LASA | 54 (9-115) | 35 (2-165) |
| Qualitator | 64 (42-127) | 75 (44-117) |
| Scores on range of 0-10 | | |
| NHP | 2.2 (0.2-5.1) | 1.5 (0.2-5.8) |
| LASA | 2.5 (0.4-5.3) | 1.6 (0.1-7.6) |
| Qualitator | 2.8 (0.7-8.7) | 3.8 (0.9-7.8) |

Correspondence: S.C.A. Fraser, Department of Surgery, Kings College Hospital, Denmark Hill, London SE5 9RS, UK.
Received 14 March 1992; and in revised form 23 September 1992.

phosphamide 100 mg $m^{-2}$ orally on days 1–14, Methotrexate 35 mg $m^{-2}$ intravenously on days 1 and 8 and 5-fluorouracil 600 mg $m^{-2}$ intravenously on days 1 and 8, on a 28 day cycle.

Anti-emetics were given parenterally or orally as appropriate. In practice, intravenous Metaclopramide 10 mg was given prophylactically to every patient receiving CMF at the time of cytotoxic administration and Prochloperazine in tablet or suppository from was given on request to patients to take at home. Dose reductions were made for patients over 65 years old and dose modification made if the WBC fell below 3000 $l^{-6}$ or platelets to below 100 $l^{-6}$. One patient on CMF experienced mucositis for which she was given Calcium Folinate 15 mg every 6 h for 24 h.

*Assessment of disease*

The endpoints chosen were: Time to treatment failure, survival, International Union Against Cancer (UICC) response criteria, World Health Organisation (WHO) toxicity criteria and Quality of Life. Time to treatment failure was defined as the time to progression of lesions either on measurement or symptomatically requiring addition to or alteration in therapy, or the abandonment of treatment due to toxicity. If treatment failure occurred before completion of a 6-month course of treatment, alternative therapy was given as appropriate. After 6 months, chemotherapy ceased and no treatment was given until or unless recurrence occurred or disease progressed. Clinical and laboratory measurements made at entry to study were a full medical history and examination , weight, height, age, date of birth, PMH, full blood count, differential WBC, biochemical screen. Photographs were taken of visible lesions and records made of tumour dimensions. All patients had a bone scan, liver ultrasound scan and chest radiograph. CT scan was performed in patients whose lesions were not otherwise measurable. Quality of life assessment was made using the Nottingham Health Profile (Hunt *et al.*, 1985) and Linear Analogue Self Assessment (Priestman & Baum, 1976) at the start of treatment and four weekly thereafter; throughout treatment, patients completed the Qualitator daily diary card, a new instrument developed for breast cancer chemotherapy trials (Fraser *et al.*, 1990). Full blood count was measured prior to administration of intravenous cytotoxics. Patient characteristics were compared using the Chi-squared and *t*-tests.

*Survival and response analysis*

UICC criteria of response were assessed 4 weekly. The WHO toxicity criteria were recorded every 4 weeks. UICC response rates were compared using the Chi-squared test and time to treatment failure and survival analyses were done using the Kaplan-Meier life table method (Kaplan & Meier, 1958) and log rank test (Peto *et al.*, 1977). Correlation between initial QoL scores and survival were done using Spearman's rank correlation method.

*Quality of life analysis*

With all instruments, a high score indicates poor QoL. The NHP scores were analysed as recommended by the authors so that at each completion, a weighted score out of a possible 100 was obtained for each of the six components: emotional state, energy, pain, physical mobility, sleep and social factors. In this study, the components were then added to give a global score range of 0–600. The LASA questionnaire consisted of 26 categories, each scored 0–9 on a visual analogue scale. Two categories, the 'open' item and the general statement on QoL were excluded from analysis, as the former was ignored by most patients and the latter was judged to duplicate the rest of the questionnaire. The global range was therefore 0–216. Both NHP and LASA were compared between patient groups at each juncture using the Mann-Whitney-U test. Comparison with subsequent scores was performed using the Wilcoxon rank test. Completion of the

**Figure 1** Time to treatment failure (medians CMF 24 weeks, Epirubicin 7 weeks, $\chi^2 = 5.17$, 1 d.f., $P<0.05$, Epirubicin $n = 21$, CMF $n = 19$).

Qualitator involves the choice of five symptoms from a menu of 23, in four domains, scoring on a categorical scale 1–4. The details are described in the accompanying paper in this issue; the range of the weekly global scores is from 35–140. For comparison, pre-treatment NHP and LASA scores were compared with the first week of the Qualitator and thereafter, the comparison of NHP and LASA 4 weekly scores was with an average of each patient's aggregated Qualitator scores for that period. Analysis was then performed using the same non-parametric methods as for the NHP and LASA. Analysis of individual Qualitator symptoms is also described in the accompanying paper.

*Exclusions*

Forty patients were entered into the trial. Thirty-seven patients completed the NHP and 36 the LASA at the start of the study. Three exclusions were patients who were unable to start treatment following randomisation and subsequently left the study. The other LASA was incorrectly completed by the fourth patient. Thereafter, patients remaining in the study completed the NHP and LASA during each month of treatment. Three CMF patients failed to do so at 1 month and one at 5 months; one patient failed to complete them at 4 months. The Qualitator was commenced by 29 patients. At the start of the study three elderly patients were, in retrospect mistakenly, not offered the Qualitator. One patient, once randomised refused to complete it, one progressed rapidly after 1 month and was unable to return the card. The remaining six patients progressed rapidly within a week of the start of treatment and were also unable to return the diary cards.

### Results

*UICC response*

The response rates according to UICC criteria were 58% for the CMF group and 29% for the Epirubicin group ($\chi^2 = 3.51$, 1 d.f., $P>0.05$, see Table II). If the six patients who relapsed before or within the first week of treatment are

**Table II** Response by randomisation

| *UICC response* | *CMF* | *Epirubicin* |
| --- | --- | --- |
| Complete | 1 | 0 |
| Partial | 10 | 6 |
| No change | 2 | 7 |
| Progression | 3 | 5 |
| Rapid progression | 3 | 3 |

$\chi^2 = 3.510$, 1 d.f., $P>0.05$. Excluding 3 in each group with rapidly progressive disease, $\chi^2 = 4.300$, 1 d.f., $P<0.05$.

excluded as in other studies the difference is significant ($\chi^2 = 4.30$, 1 d.f., $P<0.05$).

The time to treatment failure was longer for CMF patients than epirubicin patients: median 24 weeks and 7 weeks ($\chi^2 = 5.17$, 1 d.f., $P<0.05$, see Figure 1).

*Survival*

Survival was similar in both treatment groups: medians 57 weeks and 55 weeks respectively ($\chi^2 = 1.38$; 1 d.f., $P = 0.24$) (see Figure 2). UICC responders, as expected from many previous studies (A'Hern *et al.*, 1988) survived longer than that non-responders: medians 87 weeks and 30 weeks ($\chi^2 = 5.42$, 1 d.f., $P<0.05$, see Figure 3).

**Figure 2** Overall survival (medians CMF 57 weeks, Epirubicin 55 weeks, $\chi^2 = 1.38$, 1 d.f., $P = 0.24$, Epirubicin $n = 21$, CMF $n = 19$).

**Figure 3** Overall survival according to UICC response (medians responders 87 weeks, non-responders 30 weeks, $\chi^2 = 5.42$, 1 d.f., $P = 0.02$, response $n = 17$, no response $n = 23$).

**Table III** Toxicity by WHO grade: number (%) of each treatment group in each category, on each month of treatment

| *Rx* | *Total* | *WHO grade* | *Alopecia* | *Nausea or vomiting* | *Haematological* |
|---|---|---|---|---|---|
| Epi | 83 | 0 | 75 (90) | 83 (100) | 82 (99) |
| | | 1 | 8 (10) | 0 | 0 |
| | | 2 | 0 | 0 | 0 |
| | | 3/4 | 0 | 0 | 1 (1) |
| CMF | 106 | 0 | 43 (41) | 64 (60) | 75 (71) |
| | | 1 | 20 (19) | 22 (21) | 20 (19) |
| | | 2 | 12 (11) | 10 (9) | 7 (7) |
| | | 3/4 | 31 (29) | 10 (9) | 4 (4) |
| | | | $P<0.001$ | $P<0.001$ | $P<0.02$ |

*Toxicity*

Toxicity was very low for all patients receiving epirubicin. CMF caused significantly more alopecia ($P<0.001$), nausea and vomiting ($P<0.001$) and haematological toxicity ($P<0.02$) above WHO grade I (see Table III). One CMF patient required hospital admission for treatment of septicaemia. One epirubicin patient receiving prednisolone for scleroderma developed septicaemia requiring hospital admission. There were no fatalities due to side-effects of treatment.

*Quality of life at entry to the trial*

The respective NHP, LASA and equivalent aggregated weekly Qualitator scores were compared for each 4 weeks. Patients' QoL scores were analysed according to response and to treatment. Prior to the start of treatment, a poorer QoL was recorded amongst patients who subsequently did not respond, statistically significant only for the LASA, ($P<0.002$). The pre-treatment scores are illustrated in Figure 4, in which the LASA, NHP and Qualitator scores are standardised to a scale of 0–10.

Patients' QoL scores at the start of the study were correlated by rank with their subsequent survival. The Spearman co-efficients were −0.52 (95% c.i., −0.72, −0.23) for the LASA, −0.35 (−0.60, 0.04) for the NHP, −0.64 (−0.82, −0.36) for the Qualitator.

*Quality of life during treatment*

Compliance for the 29 patients who started the Qualitator, the 37 who started the NHP and 36 who started the LASA respectively were 88%, 89% and 92%. Figure 5 shows the mean global QoL values in each treatment group at each stage for all patients remaining in the study. The means are used purely for graphic representation: statistical comparison between treatment groups was by a rank test at each 4 weeks.

By 3 months, the scores of patients with a UICC response in both the epirubicin and CMF treatment groups had improved significantly in the Qualitator (medians 60.5 to 48, $P<0.05$) and NHP (medians 83 to 24, $P<0.05$) though not in the LASA (medians 22 to 29) scores (see Figures 6a to 6c). Nonresponders experienced no significant difference in their initial scores and the final scores prior to treatment failure: Qualitator medians 80 to 74 ($P = 0.5$), NHP medians 133 to 182 ($P = 0.435$), LASA medians 64 to 71 ($P = 0.55$). The

**Figure 4** Standardised QoL scores at start of treatment according to subsequent response: Qual = Qualitator, NHP = Nottingham Health Profile, LASA = Linear Analogue Self-Assessment, Res = Response, No R = No Response.

**Figure 5** Standardised mean QoL scores at each month of treatment for Epirubicin patients ——— and CMF patients ·······.

pretreatment difference in scores between responders and non-responders persisted on each monthly comparison: one month (LASA $P<0.02$, NHP $P<0.01$, Qualitator $P<0.05$), 2 months (Qualitator $P<0.05$), 3 months (NHP $P<0.05$, Qualitator $P<0.01$) and 4 months (NHP $P<0.05$).

All of the QoL measures allow sub-analysis in considerable detail. In separate analysis of the six domains of the NHP (emotional state, energy, pain, physical mobility, sleep and social factors) and the LASA and Qualitator symptoms in four sub-groups (physical symptoms, social factors, emotional factors and physical performance), non-responders had worse scores at most stages (see accompanying paper in this issue). The only significant difference between treatment groups was a better score in CMF than Epirubicin patients in the NHP score for pain at 2 months (median differences 0 and 9.5, $P<0.05$), energy at 3 months (medians 0 and 24, $P<0.05$) and a worse Qualitator score at 3 months for personal relationships in CMF patients (median 7 and 7.65, $P<0.05$). In each case the high scores were amongst the non-responders in each group.

### Discussion

One of the most difficult decisions facing clinicians treating patients with advanced breast cancer is what to do when second line hormone therapy fails. At what point does one advise chemotherapy, to whom and how aggressively? Until recent years, the success of a treatment regimen has been defined almost solely by tumour shrinkage. Although toxic side effects have been measured, there was little evidence of correlation with the patient's experience. The failure of many studies to show a survival advantage to any regimen caused some clinicians to question the merits of giving chemotherapy at all (Powles *et al.*, 1980). During the last decades, the concept of Quality of Life has become increasingly important in those patients in whom little survival advantage is anticipated through treatment and efforts were made to define and measure it (Fallowfield, 1990). Increasing numbers, but still a minority, of studies measure QoL (Bryne, 1992). The disparate instruments and periods of measurement have made it difficult to interpret how chemotherapy affects QoL for patients with advanced breast cancer. The aim of this study was to compare a standard combination regimen with a single agent regimen in which different toxicity and possibly different response rates could be anticipated, and whether a difference in survival or QoL would result. Detailed intermittent QoL measurement was made with three instruments, two of which were specifically designed for the task. The response data were consistent with previous studies in that the patients who had a measurable response enjoyed longer overall survival. Although survival among patients with non-progressive disease was better for CMF patients, the poor survival of CMF non-responders was enough to redress this balance so

**Figure 6** a,b,c: Standardised QoL scores in patients who responded, at 1 week and 12 weeks for the Qualitator (15 patients), before treatment and at 3 months for the Nottingham Health Profile (17 patients) and Linear Analogue Self-Assessment (17 patients).

that survival for the two treatment groups as a whole was equal. Few studies are large enough to show a survival difference between treatment groups, but A'Hern et al. showed that a better response rate equated with longer median survival in a statistical overview of 50 chemotherapy trials (A'Hern et al., 1988). The QoL data were not wholly expected. Although Ebbs et al. (1988) had reported that good pre-treatment QoL scores were associated with a subsequent response, we found that there was a close correlation with subsequent duration of survival too. Morris and Sherwood (1987) described this in terminally ill patients, and Addington-Hall et al. (1990) used the Spitzer QoL Index (Spitzer et al., 1981) to predict duration of survival in 230 terminally ill patients. However, it was a surprise that even in this small study, such a consistent trend would emerge. In the context of patients with advanced breast cancer, this may be of significance in deciding on treatment.

Low objective toxicity in patients treated with epirubicin was reflected in the recording of specific treatment-related symptoms in the Qualitator, but QoL scores overall were unaffected and resembled closely the global scores of the other two instruments.

Is a harsher regimen therefore the treatment of choice for advanced breast cancer? The evidence is that it does not impair QoL in non-responders of whom there are fewer anyway and QoL improves for responders, as previously reported by Baum et al. (1980). Coates et al. (1987) found that Quality of Life declined significantly in patients on a less aggressive regimen in which response was poorer and Slevin et al. (1990) found cancer patients much more willing to comtemplate radical chemotherapy than were their doctors for them. However, if pre-treatment QoL scores give not only a guide to response, but to survival as well, then perhaps those patients with clinically advanced disease in whom QoL is poor, who will not respond and whose survival will be poor should not be given chemotherapy at all. A different interpretation might be that those patients whose disease is not yet advanced enough to affect their QoL are those most likely to respond to treatment. In a recent study, patients with metastatic cancer of bowel, lung, pancreas or melanoma, who received conventional therapy, including chemotherapy, had no better survival than matched controls having 'alternative' therapy. Chemotherapy was not associated with a *worse* QoL, and although the change in QoL was similar in both groups, the patients treated conventionally started and finished with better QoL measurement. This may have been influenced by the social composition of the groups: a higher number of alternative therapy patients had degrees and poor QoL may have contributed to their decision to seek unproven therapy. The ideal study in such patients would be randomised, with an arm involving palliative care only (Cassileth et al., 1991).

One way of resolving the difficulty would be to involve the patient more fully in the decision-making process. This approach was recently advocated in early breast cancer treatment by Wennberg and colleagues who have used interactive videotapes (Wall Street Journal, 1992).

The present study does not provide solutions to these uncertainties. However, detailed QoL measurement is shown to add valuable and perhaps not wholly expected information in evaluating advanced breast cancer chemotherapy. QoL measurement may be of use in defining individual strategies. Only by including QoL measurement in more protocols will knowledge of its precise role become clear.

We are most grateful to Farmitalia Carlo Erba for the financial and technical support which enabled this study to proceed.

### References

ADDINGTON-HALL, J.M., MACDONALD, L.D. & ANDERSON, H.R. (1990). Can the Spitzer Quality of Life Index help to reduce prognostic uncertainty in terminal care? *Br. J. Cancer*, **62**, 695-699.

A'HERN, R.P., EBBS, S.R. & BAUM, M. (1988). Does chemotherapy improve survival in advanced breast cancer? A statistical overview. *Br. J. Cancer*, **57**, 615-618.

BAUM, M., PRIESTMAN, T., WEST, R.R. & JONES, E.M. (1980). A comparison of subjective responses in a trial comparing endocrine with cytotoxic treatment in advanced carcinoma of the breast. *Eur. J. Cancer* (supplement 1): 223-226.

BONADONNA, G. & VAN OOSTEROM, A. (1983). Treatment of advanced breast cancer; workshop report. *Eur. J. Cancer & Clin. Oncol.*, **19**, 1779-1781.

BYRNE, M. (1992). Cancer chemotherapy and quality of life. *Br. Med. J.*, **304**, 1523-1524.

CASSILETH, B.R., LUSK, E.J., GUERRY, DUP., BLAKE, A.D., WALSH, W.P., KASCIUS, L. & SCHULTZ, D.J. (1991). Survival and quality of life among patients receiving unproven as compared with conventional cancer therapy. *New Engl. J. Med.*, **324**, 1180-1185.

CHLEBOWSKI, R.T., SMALLEY, R.V., WEINER, J.M., IRWIN, L.E., BARTOLUCCI, A.A. & BATEMAN, J.R. (1989). Combination *versus* sequential single agent chemotherapy in advanced breast cancer: associations with metastatic sites and long-term survival. *Br. J. Cancer*, **59**, 227-230.

COATES, M.D., GEBSKY, V., STAT, M., BISHOP, J.F., JEAL, P.N., WOODS, R.L., SNYDER, R., TATTERSALL, M.H.N., BYRNE, M., HARVEY, V., GILL, G., SIMPSON, J., DRUMMOND, R., BROWNE, J., VAN COOTEN, R. & FORBES, J.F. (1987). Improving the quality of life during chemotherapy for advanced breast cancer. A comparison of continuous and intermittent treatment strategies. *New Eng. J. Med.*, **317**, 1490-1495.

EBBS, S.R., SAUNDERS, J.A., GRAHAM, H., A'HERN, R.P., BATES, T. & BAUM, M. (1989). Advanced breast cancer: a randomised trial of epirubicin at two different dosages and two administration systems. *Acta Oncol.*, **28**, 887-891.

EBBS, S.R., A'HERN, R.P., GRAHAM, H. & BAUM, M. (1988). Subjective measurements of quality of life predict response to chemotherapy for advanced breast cancer (abstract). *Br. J. Surg.*, **75**, 601.

FALLOWFIELD, L.J. (1990). *The Quality of Life – The Missing Measurement in Health Care*. Souvenir Press: London.

FRASER, S.C.A., EBBS, S.R., DOBBS, H.J., FALLOWFIELD, L.J. & BAUM, M. (1990). The design of advanced breast cancer trials – new approaches. *Acta Oncol.*, **29**, 397-400.

HARRIS, A.L., CANTWELL, M.J., CARMICHAEL, J., WILSON, R., FARNDON, J., DAWES, P., GHANI, S. & EVANS, R.G.B. (1990). Comparison of short-term and continuous chemotherapy (mitozantrone) for advanced breast cancer. *Lancet*, **335**, 186-190.

HUNT, S.M., MCEWEN, J. & MCKENNA, S.P. (1985). Measuring health status: a new tool for clinicians and epidemiologists. *J. Roy. Coll. Gen. Practit.*, **35**, 185-188.

JONES, W.G. (1988). Weekly low-dose epirubicin: effective single agent therapy for advanced breast cancer without significant toxicity. Unpublished study 1988.

KAPLAN, E.L. & MEIER, P. (1958). Non-parametric oestimation from incomplete observation. *J. Am. Statist. Assoc.*, **53**, 451.

MORRIS, J.N. & SHERWOOD, S. (1987). Quality of life cancer patients at different stages of the disease trajectory. *J. Chron. Dis.*, **40**, 545-553.

PETO, R., MIKE, M.C., ARMITAGE, P., BRESLOW, N.E., COX, D.R., HOWARD, S.V., MANTEL, N., MCPHEARSON, K., PETO, J. & SMITH, P.G. (1977). Design and analysis of randomised clinical trials requiring prolonged observations of each patient. Part 2. Analysis and examinations. *Br. J. Cancer*, **35**, 1.

POWLES, T.J., COOMBES, R.C., SMITH, I.E., JONES, J.M., FORD, H.T. & GAZET, J.-C. (1980). Failure of chemotherapy to prolong survival in a group of patients with metastatic breast cancer. *Lancet* **i**, 580-582.

PRIESTMAN, T.J. & BAUM, M. (1976). Evaluation of quality of life in patients receiving treatment for advanced breast cancer. *Lancet*, **?**, 899-901.

SLEVIN, M.L., STUBBS, L., PLANT, H., WILSON, P., GREGORY, W.M., ARMES, P.J. & DOWNER, S.M. (1990). Attitudes to chemotherapy: comparing views of patients with cancer with those of doctors, nurses and general public. *Br. Med. J.*, **300**, 1458-1460.

SPITZER, W.O., DOBSON, A.J., HALL, J., CHESTERMAN, E., LEVI, J., SHEPHERD, R., BATTISTA, R.N. & CATCHLOVE, B.R. (1981). Measuring the quality of life of cancer patients. A concise QL-Index for use by physicians. *J. Chron. Dis.*, **34**, 585-597.

WALL STREET JOURNAL (1990). February 1992, bl-b2.


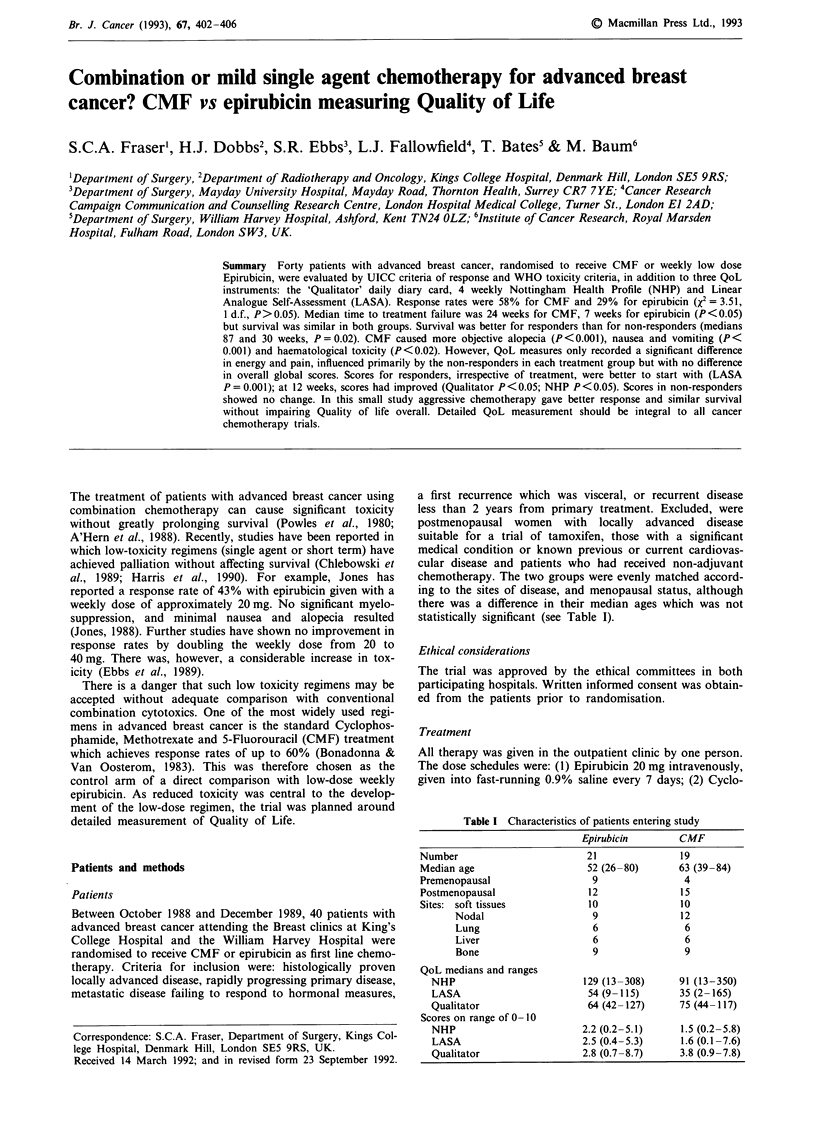

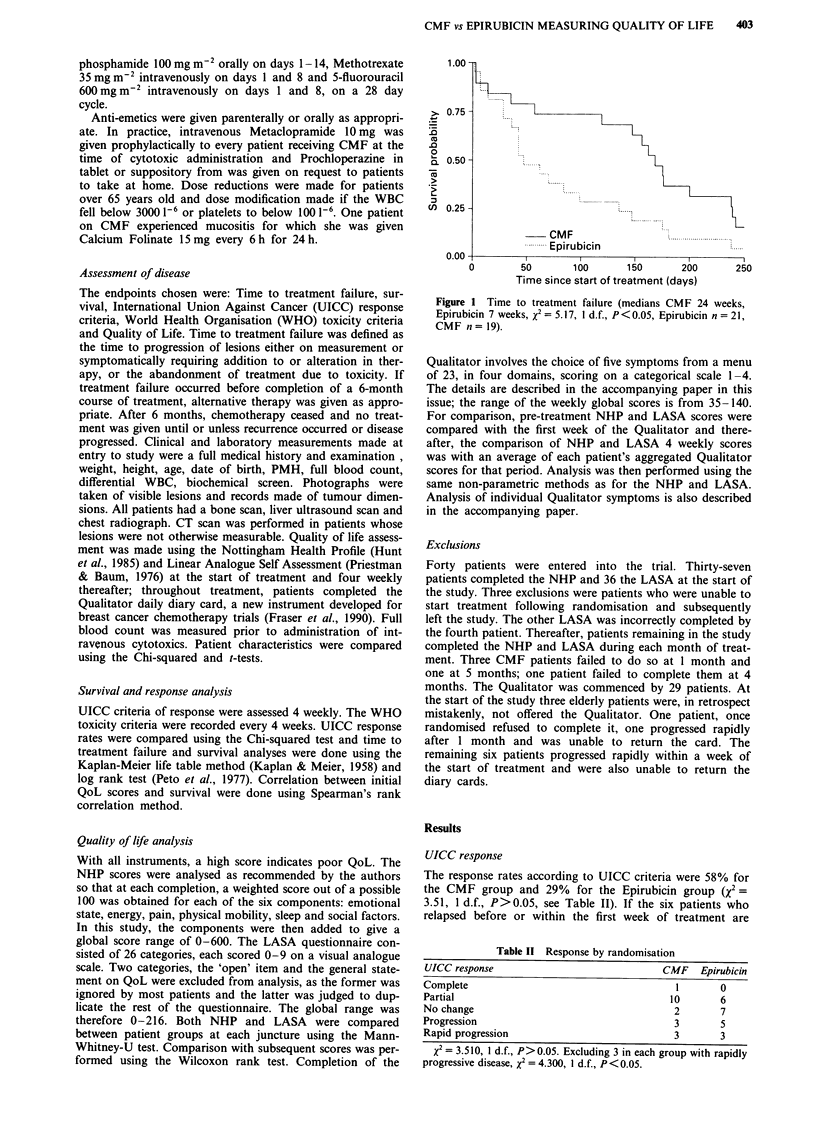

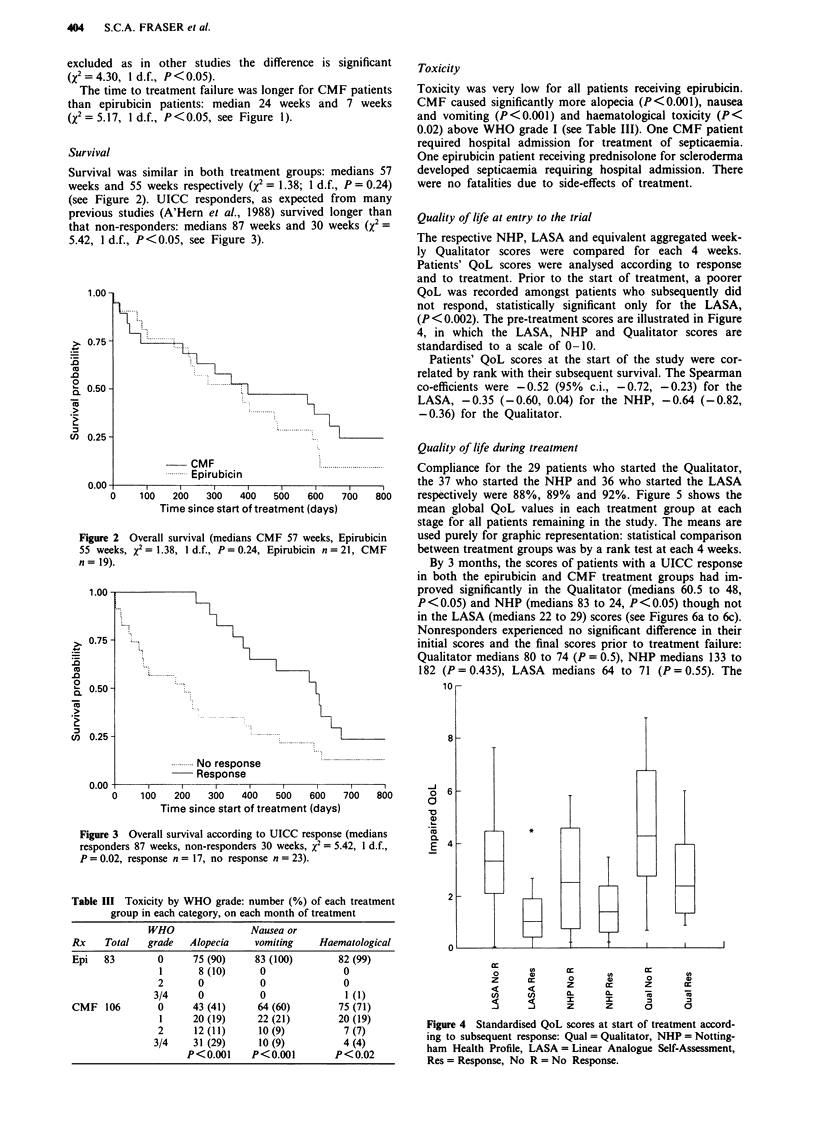

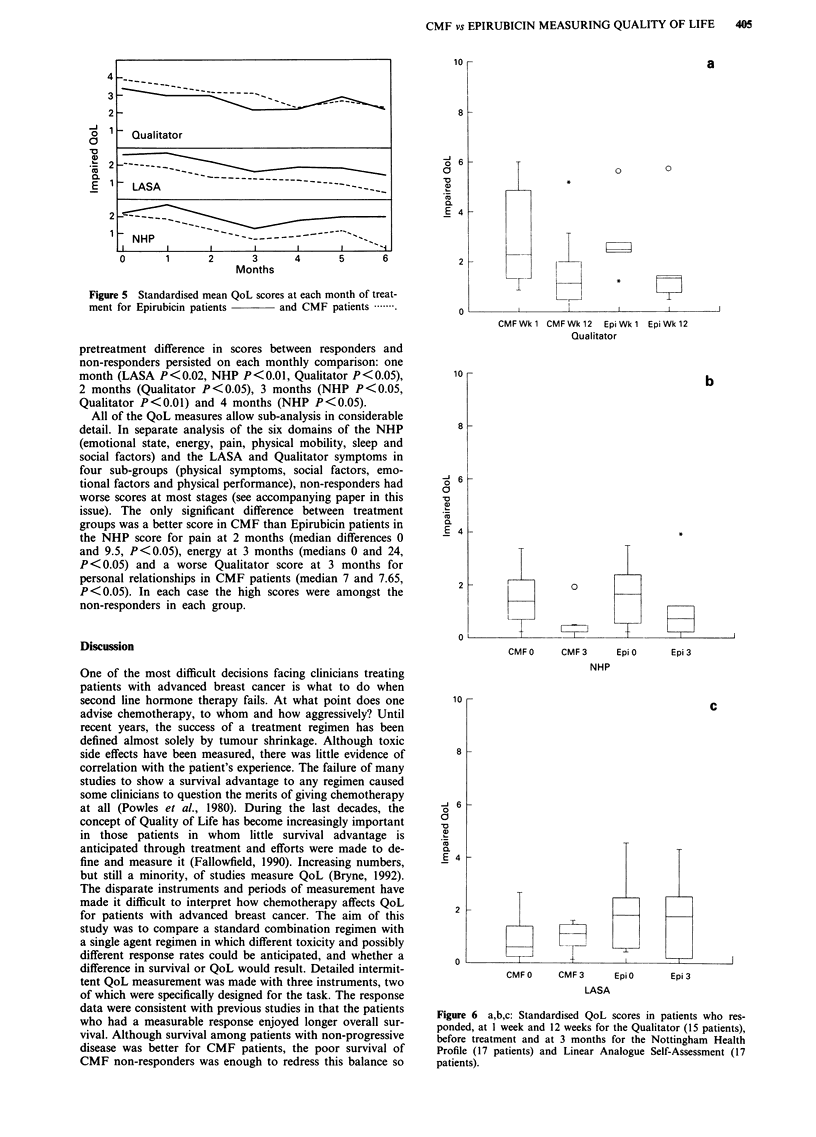

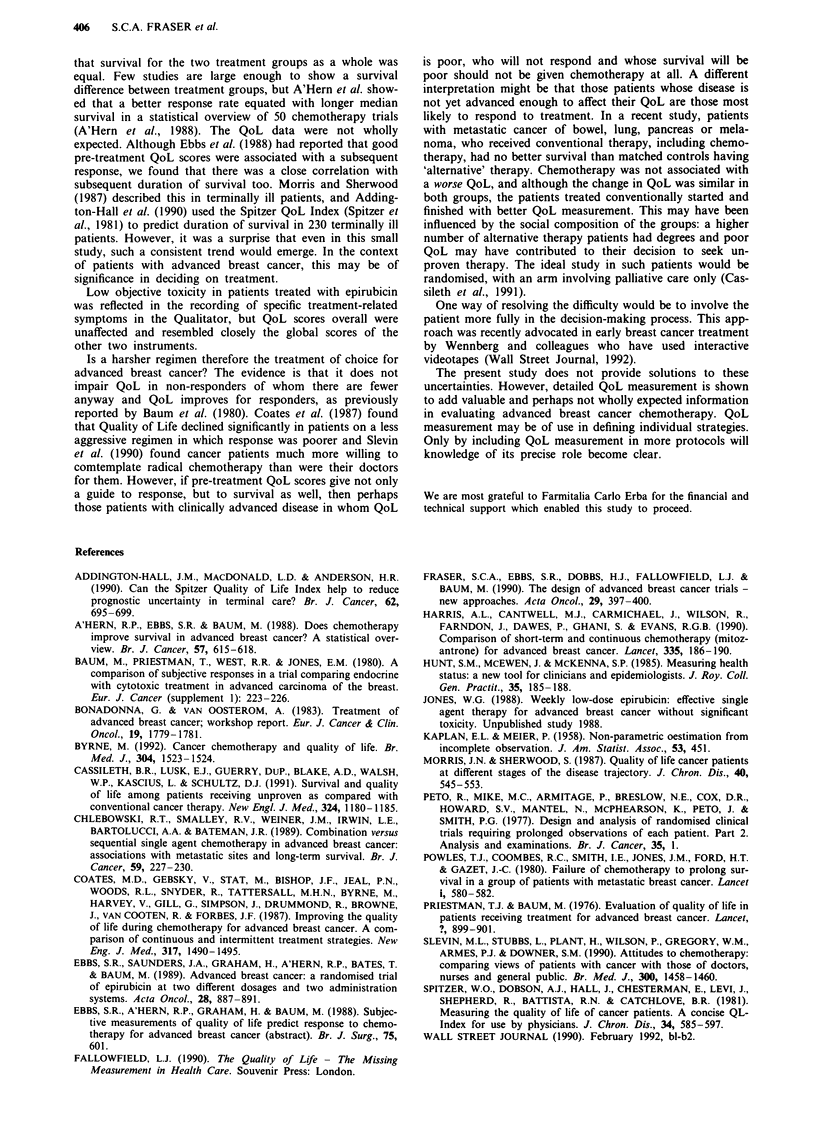


## References

[OCR_00777] A'Hern R. P., Ebbs S. R., Baum M. B. (1988). Does chemotherapy improve survival in advanced breast cancer? A statistical overview.. Br J Cancer.

[OCR_00771] Addington-Hall J. M., MacDonald L. D., Anderson H. R. (1990). Can the Spitzer Quality of Life Index help to reduce prognostic uncertainty in terminal care?. Br J Cancer.

[OCR_00782] Baum M., Priestman T., West R. R., Jones E. M. (1980). A comparison of subjective responses in a trial comparing endocrine with cytotoxic treatment in advanced carcinoma of the breast.. Eur J Cancer.

[OCR_00793] Byrne M. (1992). Cancer chemotherapy and quality of life.. BMJ.

[OCR_00797] Cassileth B. R., Lusk E. J., Guerry D., Blake A. D., Walsh W. P., Kascius L., Schultz D. J. (1991). Survival and quality of life among patients receiving unproven as compared with conventional cancer therapy.. N Engl J Med.

[OCR_00802] Chlebowski R. T., Smalley R. V., Weiner J. M., Irwin L. E., Bartolucci A. A., Bateman J. R. (1989). Combination versus sequential single agent chemotherapy in advanced breast cancer: associations with metastatic sites and long-term survival. The Western Cancer Study Group and The Southeastern Cancer Study Group.. Br J Cancer.

[OCR_00809] Coates A., Gebski V., Bishop J. F., Jeal P. N., Woods R. L., Snyder R., Tattersall M. H., Byrne M., Harvey V., Gill G. (1987). Improving the quality of life during chemotherapy for advanced breast cancer. A comparison of intermittent and continuous treatment strategies.. N Engl J Med.

[OCR_00818] Ebbs S. R., Saunders J. A., Graham H., A'Hern R. P., Bates T., Baum M. (1989). Advanced breast cancer. A randomised trial of epidoxorubicin at two different dosages and two administration systems.. Acta Oncol.

[OCR_00834] Fraser S. C., Ebbs S. R., Dobbs H. J., Fallowfield L. J., Baum M. (1990). The design of advanced breast cancer trials. New approaches.. Acta Oncol.

[OCR_00839] Harris A. L., Cantwell B. M., Carmichael J., Wilson R., Farndon J., Dawes P., Ghani S., Evans R. G. (1990). Comparison of short-term and continuous chemotherapy (mitozantrone) for advanced breast cancer.. Lancet.

[OCR_00845] Hunt S. M., McEwen J., McKenna S. P. (1985). Measuring health status: a new tool for clinicians and epidemiologists.. J R Coll Gen Pract.

[OCR_00859] Morris J. N., Sherwood S. (1987). Quality of life of cancer patients at different stages in the disease trajectory.. J Chronic Dis.

[OCR_00864] Peto R., Pike M. C., Armitage P., Breslow N. E., Cox D. R., Howard S. V., Mantel N., McPherson K., Peto J., Smith P. G. (1977). Design and analysis of randomized clinical trials requiring prolonged observation of each patient. II. analysis and examples.. Br J Cancer.

[OCR_00871] Powles T. J., Coombes R. C., Smith I. E., Jones J. M., Ford H. T., Gazet J. C. (1980). Failure of chemotherapy to prolong survival in a group of patients with metastatic breast cancer.. Lancet.

[OCR_00877] Priestman T. J., Baum M. (1976). Evaluation of quality of life in patients receiving treatment for advanced breast cancer.. Lancet.

[OCR_00882] Slevin M. L., Stubbs L., Plant H. J., Wilson P., Gregory W. M., Armes P. J., Downer S. M. (1990). Attitudes to chemotherapy: comparing views of patients with cancer with those of doctors, nurses, and general public.. BMJ.

[OCR_00888] Spitzer W. O., Dobson A. J., Hall J., Chesterman E., Levi J., Shepherd R., Battista R. N., Catchlove B. R. (1981). Measuring the quality of life of cancer patients: a concise QL-index for use by physicians.. J Chronic Dis.

